# Genome-wide family prediction unveils molecular mechanisms underlying the regulation of agronomic traits in *Urochloa ruziziensis*

**DOI:** 10.3389/fpls.2023.1303417

**Published:** 2023-12-12

**Authors:** Felipe Bitencourt Martins, Alexandre Hild Aono, Aline da Costa Lima Moraes, Rebecca Caroline Ulbricht Ferreira, Mariane de Mendonça Vilela, Marco Pessoa-Filho, Mariana Rodrigues-Motta, Rosangela Maria Simeão, Anete Pereira de Souza

**Affiliations:** ^1^ Center for Molecular Biology and Genetic Engineering (CBMEG), University of Campinas (UNICAMP), Campinas, São Paulo, Brazil; ^2^ Department of Plant Biology, Biology Institute, University of Campinas (UNICAMP), Campinas, São Paulo, Brazil; ^3^ Embrapa Gado de Corte, Brazilian Agricultural Research Corporation, Campo Grande, Mato Grosso, Brazil; ^4^ Embrapa Cerrados, Brazilian Agricultural Research Corporation, Brasília, Brazil; ^5^ Department of Statistics, University of Campinas (UNICAMP), Campinas, São Paulo, Brazil

**Keywords:** feature selection, forage grasses, gene coexpression networks, genomic prediction, machine learning, major importance markers, RNA-Seq

## Abstract

Tropical forage grasses, particularly those belonging to the *Urochloa* genus, play a crucial role in cattle production and serve as the main food source for animals in tropical and subtropical regions. The majority of these species are apomictic and tetraploid, highlighting the significance of *U. ruziziensis*, a sexual diploid species that can be tetraploidized for use in interspecific crosses with apomictic species. As a means to support breeding programs, our study investigates the feasibility of genome-wide family prediction in *U. ruziziensis* families to predict agronomic traits. Fifty half-sibling families were assessed for green matter yield, dry matter yield, regrowth capacity, leaf dry matter, and stem dry matter across different clippings established in contrasting seasons with varying available water capacity. Genotyping was performed using a genotyping-by-sequencing approach based on DNA samples from family pools. In addition to conventional genomic prediction methods, machine learning and feature selection algorithms were employed to reduce the necessary number of markers for prediction and enhance predictive accuracy across phenotypes. To explore the regulation of agronomic traits, our study evaluated the significance of selected markers for prediction using a tree-based approach, potentially linking these regions to quantitative trait loci (QTLs). In a multiomic approach, genes from the species transcriptome were mapped and correlated to those markers. A gene coexpression network was modeled with gene expression estimates from a diverse set of *U. ruziziensis* genotypes, enabling a comprehensive investigation of molecular mechanisms associated with these regions. The heritabilities of the evaluated traits ranged from 0.44 to 0.92. A total of 28,106 filtered SNPs were used to predict phenotypic measurements, achieving a mean predictive ability of 0.762. By employing feature selection techniques, we could reduce the dimensionality of SNP datasets, revealing potential genotype-phenotype associations. The functional annotation of genes near these markers revealed associations with auxin transport and biosynthesis of lignin, flavonol, and folic acid. Further exploration with the gene coexpression network uncovered associations with DNA metabolism, stress response, and circadian rhythm. These genes and regions represent important targets for expanding our understanding of the metabolic regulation of agronomic traits and offer valuable insights applicable to species breeding. Our work represents an innovative contribution to molecular breeding techniques for tropical forages, presenting a viable marker-assisted breeding approach and identifying target regions for future molecular studies on these agronomic traits.

## 1 Introduction

Pastures composed of tropical forage grasses, particularly those belonging to the *Urochloa* genus, serve as the main food source for livestock animals in tropical and subtropical regions. These pastures play a significant role in the economic sectors associated with beef and dairy production, as well as seed markets (Jank et al., 2014; Ferreira et al., 2021). The genetic improvement of *Urochloa* species is recent, starting approximately 40 years ago, and presents challenges due to varying ploidy levels, high heterozygosity, and a prevalent mode of reproduction through apomixis (Ferreira et al., 2021; Simeão et al., 2021). Among the main goals of breeding programs are the development of cultivars that exhibit tolerance to biotic stresses, adaptability to future climate changes, and increased productivity with enhanced nutritional value to optimize animal performance (Pereira et al., 2018b; Simeão et al., 2021).

These goals can be expedited through the incorporation of genomic selection (GS) into breeding cycles. GS employs statistical models to perform genomic predictions (GPs) of plant performance based on genetic markers, mainly single nucleotide polymorphisms (SNPs) (Daetwyler et al., 2013). Although the estimation of GP models has already demonstrated feasibility in other important polyploid crops (de Bem Oliveira et al., 2020; Pincot et al., 2020; Ferrão et al., 2021; Haile et al., 2021; Juliana et al., 2022; Petrasch et al., 2022), this methodology has only recently started to be tested in *Urochloa* spp. (Matias et al., 2019a; Aono et al., 2022). Therefore, efforts must be directed toward the establishment of high-quality marker panels and large-scale phenotyping (Simeão et al., 2021). Fortunately, two *Urochloa* spp. genomes, specifically *U. ruziziensis* (2n=2x=18), have recently become available (Pessoa-Filho et al., 2019; Worthington et al., 2021), facilitating the identification of many SNPs with the potential to enhance the accuracy of GP analyses in *Urochloa* spp.

Traditionally, GP models employ a dense dataset of molecular markers to compute genomic estimated breeding values at the individual level (Meuwissen et al., 2001). However, in the case of *U. ruziziensis* and other forage species, such as alfalfa and ryegrass, it is a common practice to employ the family (full or half-siblings) as the basic unit for phenotyping and selection (Simeão et al., 2012; Simeão et al., 2016a; Simeão et al., 2016b; Biazzi et al., 2017; Cericola et al., 2018; Jia et al., 2018; Murad Leite Andrade et al., 2022). This practice makes the development of genome-wide family prediction (GWFP) approaches highly advantageous. By considering family groups as the measurement unit, there is a reduction in genotyping efforts, as well as the costs associated with developing GP models (Zou et al., 2016; Rios et al., 2021; Murad Leite Andrade et al., 2022). Furthermore, the implementation of GWFP can improve the predictive ability of selection, increasing the rate of genetic gains for complex traits, as demonstrated in studies on loblolly pine and alfalfa (Rios et al., 2021; Murad Leite Andrade et al., 2022).

To identify family-pool markers, sequencing approaches can be employed to generate a large number of SNP markers (Elshire et al., 2011; Poland et al., 2012). Genotyping-by-sequencing (GBS) is a cost-effective and high-throughput genotyping method that can be used to identify SNPs even in the absence of a reference genome. However, it is important to ensure a reasonable sequencing depth to minimize the occurrence of missing data points (Thakral et al., 2022). GBS has been employed in several studies on family-pool genotyping (Futschik and Schloütterer, 2010; Bélanger et al., 2016; Cericola et al., 2018; Schneider et al., 2022) due to its advantages and straightforward applicability in obtaining allele counts from sequencing reads (Byrne et al., 2013). Consequently, in the context of family-pool GP, the use of allele counts derived from GBS allows for direct inference without the need for estimating allelic dosages (Guo et al., 2018).

In addition to the application of GP models in GS approaches, family-pool markers can also be employed in genome-wide association studies (GWAS). Unlike selection-based applications, GWAS aims to identify loci that are associated with a greater extent of genetic variation, thereby enhancing the understanding of the genetic architecture underlying complex traits (Ashraf et al., 2014; Zhang et al., 2014; Fè et al., 2015). In this sense, adopting a family-based approach provides a more comprehensive perspective on the genetic variations related to the configuration of traits across different families. Once these genomic associations have been assessed, additional omics approaches can be employed to further elucidate the biological mechanisms triggered by adjacent genes and their association with the configuration of complex traits (Scossa et al., 2021).

Traditionally, data generated from various levels of biological information, such as genomics, transcriptomics, and proteomics, have been analyzed separately. However, more recently, the integration of data followed by appropriate statistical analysis has emerged as a promising approach to unravel the biological implications of different traits in humans (Yang et al., 2014), microorganisms (Borin et al., 2018; Rosolen et al., 2022), animals (Parker Gaddis et al., 2016; Mateescu et al., 2017), and plants (Francisco et al., 2021; Cardoso-Silva et al., 2022). Despite the economic importance of *U. ruziziensis* and the availability of molecular data resources, no study incorporating multiomics has been conducted on *U. ruziziensis* or any species of the *Urochloa* genus.

Although assessing different aspects, GP and GWAS possess complementary advantages, providing robust information for the identification of potential candidate genes related to agronomically important traits. Methodologies originally used for GP have been applied in GWAS to detect loci associated with the trait of interest (Goddard et al., 2016; Wang et al., 2020; Wolc and Dekkers, 2022). Conversely, association studies have demonstrated their usefulness in enhancing GP (Zhang et al., 2014; Bian and Holland, 2017; Jeong et al., 2020). To further enhance the outcomes of association and prediction studies, researchers have explored the integration of machine learning (ML) algorithms. Despite the controversial incorporation of ML in GP, with some studies highlighting its advantages (Ma et al., 2018; Waldmann et al., 2020; Aono et al., 2022) and others refuting them (Crossa et al., 2019; Montesinos-López et al., 2019; Zingaretti et al., 2020), numerous investigations consistently demonstrate that ML-based strategies incorporating feature selection (FS) techniques effectively reduce marker density. These methods not only maintain or enhance prediction accuracy but also enable the identification of polymorphisms associated with phenotypes (Li et al., 2018; Aono et al., 2020; Pimenta et al., 2021; Aono et al., 2022).

In this study, we assessed the feasibility of family-based genotyping in autotetraploid *U. ruziziensis* (2n = 4x = 36) and investigated the GWFP capability to predict biomass production and growth traits in both wet and dry seasons. We employed traditional statistical methods as well as ML algorithms to analyze the data. To enhance prediction accuracy, we employed FS strategies to identify subsets of SNP markers with increased predictive power. Furthermore, we used an ML tree-based approach to estimate the importance of these variations in prediction. The most significant markers were then used as a guide to map RNA-Seq assembled genes, which were considered putatively associated with the investigated traits. To gain a deeper understanding of the molecular mechanisms underlying the regulation of these traits in the different seasons investigated, we expanded the set of identified genes by constructing a gene coexpression network (GCN). Our study not only brings innovation to GWFP, but also proposes a means of integrating genomic and transcriptomic data. Moreover, our findings contribute to the expansion of knowledge on the biological processes influencing the investigated agronomic traits. The outcomes of this work offer valuable resources for future studies and breeding programs targeting the *Urochloa* genus.

## 2 Materials and methods

### 2.1 *Urochloa ruziziensis* phenotyping

The progenies used in this study were generated as part of the *Urochloa* breeding program of the Brazilian Agricultural Research Corporation (Embrapa) Beef Cattle (EBC), located in Campo Grande, Mato Grosso do Sul State, Brazil (20°27’S, 54°37’W, 530 m), as described by Simeão et al. (2012), Simeão et al. (2016a), Simeão et al. (2016b). In 2010, seven sexual autotetraploid-induced accessions (R30, R38, R41, R44, R46, R47 and R50) were replicated 20 times to create an open pollination randomized field organized into 26 lines and 12 columns spaced by 2 meters. In 2012, out of the 140 plants, 59 were selected to form breeding progenies and compose the experiment of the study. This selection was based on their viable seed production and flowering synchrony. A total of 1,180 individuals (20 seeds from each of the 59 plants selected) were planted in a randomized block design, with one plant per plot spaced 1.5 m apart (Simeão et al., 2016a, b). From the 59 half-sibling progenies, 50 were chosen based on the criterion of selecting the progenies with more plants that succeeded in the field.

The phenotypic evaluations were performed considering nine clippings at 15 cm height: (1) March 2012; (2) January 2013; (3) April 2013; (4) May 2013; (5) September 2013; (6) October 2013; (7) November 2013; (8) December 2013; and (9) January 2014. According to the climatological water balance assessed through the available water capacity (AWC) metric (**Supplementary Figure S1**) (Simeão et al., 2016a, b), six clippings were performed in the wet season (1-3,7-9) and three in the dry season (4-6). In addition to the nine clippings, we had a total sum (T) evaluation for each phenotype in the period.

**Figure 1:**
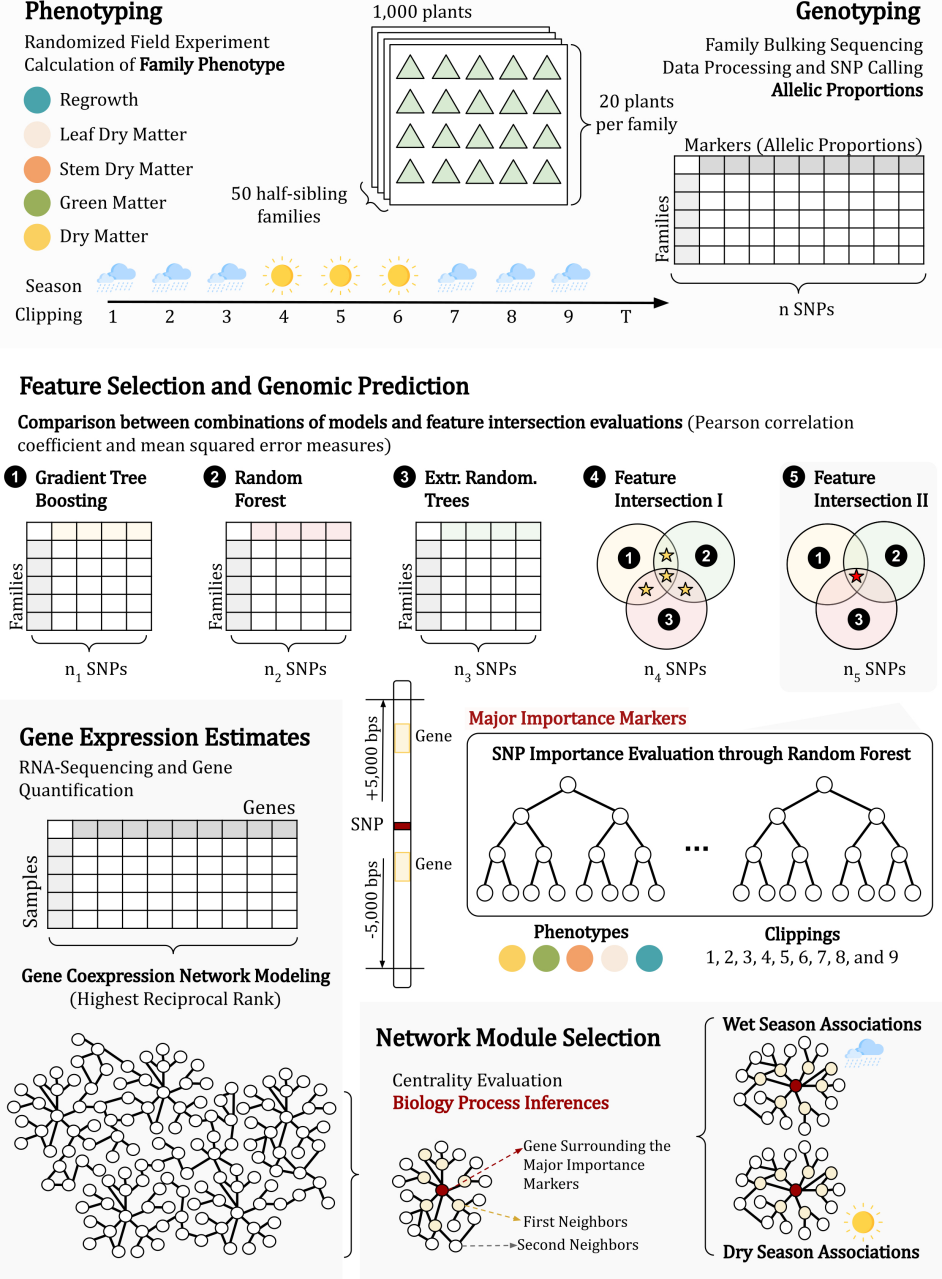
The approach established in this research can be divided into three main parts: (i) phenotyping and genotyping the population (1); (ii) identifying phenotypically associated markers through genomic prediction (2 and 3); and (iii) investigating the genes physically linked to the markers in a coexpression network (4, 5 and 6).

The agronomic traits evaluated in all clippings were green matter yield (GM) and dry matter yield (DM), both measured in grams per family, and regrowth (RG), with scores varying from 0 to 6 as described by Figueiredo et al. (2012). In addition, in clippings 2 and 5, approximately 200 g of leaves and stems from each plant were used to estimate leaf dry matter yield (LDM) and stem dry matter yield (SDM). Considering that clipping 1 was discarded from the analysis, we evaluated 33 combinations of agronomic traits and clippings (clippings 2-9 for GM, DM and RG, and clippings 2 and 5 also for SDM and LDM) (**Figure 1**), which we considered different phenotypes.

For each combination of agronomic traits and clippings, we employed the following linear mixed-effects model:


y=Xr+Zg+e

where *y* represents the phenotypic measurements, X is the design matrix of the fixed repetition effects *r*, Z is the design matrix of the random genotypic effects *g*, and *e* is the random residual vector. All the statistical analyses were performed using the software Selegen - REML/BLUP (Resende, 2002; Colombari-Filho et al., 2013). Narrow-sense trait heritability estimates were corrected using the Wright’s coefficient of relationship, as described by Simeão et al., (2016a), Simeão et al., (2016b).

To obtain family measurements, we calculated the average of each trait per family and scaled the results between 0 and 1 with the Min-Max technique. To perform a data descriptive analysis of family traits, we used boxplots to assess the distribution and outliers, computed Pearson’s correlation among all phenotype clippings and performed a principal component analysis (PCA) to assess population structure. The descriptive analysis was performed in R (R Core Team, 2021), and all PCA and plots were performed with the package pcaMethods (Stacklies et al., 2007) and the package ggplot2 (Wickham and Chang, 2016), respectively.

### 2.2 Genotyping

Genomic DNA of all individuals was extracted using the DNeasy Plant kit (QIAGEN) and pooled according to each family, totaling 50 samples. GBS libraries were constructed following the method proposed by Poland et al. (2012) using a combination of a rare cutting enzyme (EcoT22I) and a frequent cutting enzyme (MspI). Subsequently, libraries were sequenced as 150-bp single-end reads using the High Output v2 Kit (Illumina, San Diego, CA, USA) in a NextSeq 500 platform (Illumina, San Diego, CA, USA). The raw sequence data have been submitted to the NCBI Sequence Read Archive (SRA) under accession number PRJNA973612.

We performed quality evaluation of GBS raw sequence reads using FastQC version 0.11.5 (Andrews, 2010) and SNP calling using the TASSEL-GBS pipeline (Glaubitz et al., 2014) modified for polyploids (Pereira et al., 2018a). The reads were aligned to the *U. ruziziensis* genome assembly (Pessoa-Filho et al., 2019; GenBank Assembly GCA_015476505.1) using the BowTie 2.3.1 aligner (Langmead and Salzberg, 2012), and only uniquely mapped reads were employed. SNP markers were filtered using VCFtools v0.1.17 (Danecek et al., 2011) with the following criteria: a minimum sequencing depth of 20 reads, no more than 25% missing data per site, biallelic SNPs only, and removal of redundant (same genotypes in all samples) markers from the sets (**Figure 1**).

The allele frequency for each marker was estimated as the ratio between the number of reads for the alternative allele and the total number of reads. Missing data were replaced by the site mean of allele frequency. Furthermore, a PCA was performed on the complete genotype data to assess population structure.

### 2.3 Genomic prediction and feature selection

To create subsets of markers for each phenotype, three FS techniques were applied to the SNP data using the Python 3 library scikit-learn v1.0.2 (Pedregosa et al., 2011): gradient tree boosting (FS-1) (Chen & Guestrin, 2016), extremely randomized trees (FS-2) (Geurts et al., 2006), and random forest (FS-3) (Breiman, 2001). For the FS-1 technique, we employed the mean squared error (MSE) as the loss function, set the learning rate to 0.1, and considered 100 boosting stages. The criterion for assessing split quality was based on the MSE with improvement score by Friedman. We established that a minimum of 2 samples was required to split an internal node, while a minimum number of 1 sample was required for a leaf node. Furthermore, we constrained the maximum number of nodes within the trees to 3. For FS-2 and FS-3, the forest consisted of 100 trees, employing the MSE as the quality measurement function. The minimum number of samples required to split an internal node and the minimum number of samples required to form a leaf node were consistent with those of FS-1. In FS-3, the trees had no node limit, and bootstrapping was employed. Then, to perform modeling, we created feature intersection (FI) datasets by evaluating the intersection of the FS methods, considering markers that were selected by at least two FS techniques (FI-1) and markers that were selected by all three FS techniques (FI-2), similar to the approach proposed by Aono et al. (2020) and Aono et al. (2022) (**Figure 1**).

**Figure 2:**
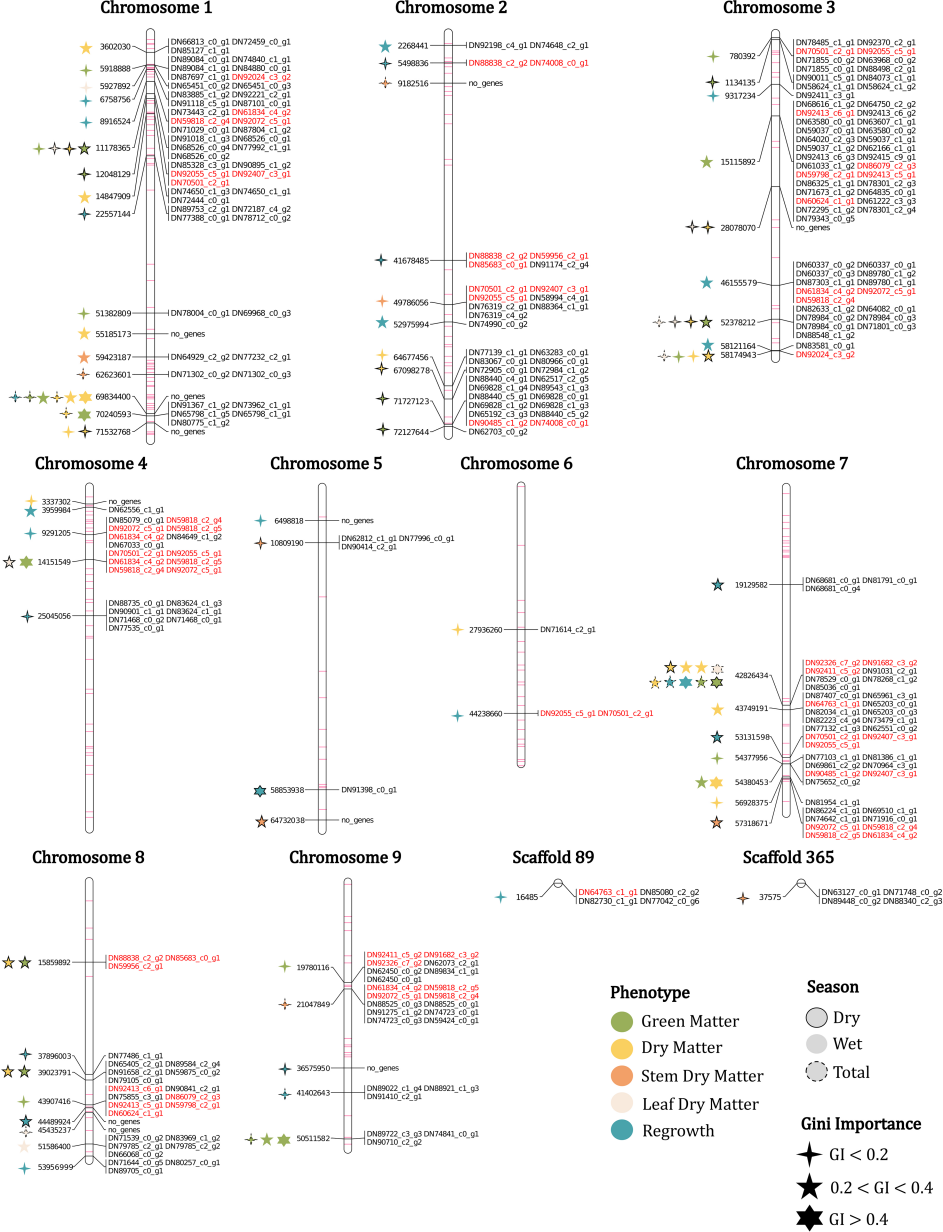
Physical map with the markers and genes associated with the phenotypes evaluated in the *U. ruziziensis* population, with Gini importance (GI) and season indicated. Duplicated genes and minor importance markers (FI-2) mapped are represented in red and purple, respectively.

As GP strategies, we estimated different models considering six regression approaches across the 33 combinations of traits and clippings, as well as both the reduced (FI-1 and FI-2) and complete versions of the dataset. As conventional GP models, we employed the semiparametric reproducing kernel Hilbert space (RKHS) with a Gaussian kernel (GK) as the covariance function using the R package BGGE v0.6.5 (Granato et al., 2018) and Bayesian ridge regression (BRR) with the R package BGLR v1.0.9 (Perez and de los Campos, 2014). Both models were estimated using 20,000 iterations with a thinning of 5 and a burn-in of 2,000. Additionally, we evaluated four ML algorithms using Python 3 with the scikit-learn library v1.0.2 (Pedregosa et al., 2011): (i) support vector machine (SVM) (Cristianini and Shawe-Taylor, 2000); (ii) random forest (RF) (Breiman, 2001); (iii) adaptive boosting (AB) (Freund and Schapire, 1997); and (iv) multilayer perceptron (MLP) neural network (Popescu et al., 2009). For SVM regression, a radial basis function was used as the kernel, with the gamma coefficient defined as $1/\left(p\times \sigma_{Z}^{2}\right)$ , where *p* represents the number of loci and $\sigma_{Z}^{2}$ the variance of the genotype matrix Z. The RF regression was performed with the same parameters as those described for FS-3. For AB, we employed a decision tree regressor as the base estimator, used a linear loss function to assign weights, and limited the maximum boosting interaction to 50 estimators. Finally, the MLP neural network was constructed with a single hidden layer comprising 100 neurons activated by the rectified linear unit (ReLU) function. We employed a quasi-Newton method to optimize the weights and applied a regularization term of 0.001 strength in the L2 regularization term.

The evaluation of the previously described models for GP was performed using a k-fold (k=5) cross validation strategy, repeated 100 times. Two metrics were measured: predictive ability (PA), quantified as the Pearson correlation coefficient, and MSE (**Figure 1**).

To compare the models, the phenotype clippings and the datasets, we used ANOVAs with multiple comparisons by Tukey’s tests implemented in the agricolae R package (De Mendiburu and De Mendiburu, 2020) (**Figure 1**). For PA, we considered the best scenario to be that in which Tukey’s test had “a” or “a” combined with other letters, such as “ab” or “abc”, which represents the highest values. On the other hand, for MSE, a scenario is better when its MSE value is lower. Therefore, we considered the best scenarios those with the higher letter or combined with other letters (i.e., “f”, “ef” or “def”).

### 2.4 Major importance markers

After identifying the best dataset of markers for each phenotype clipping, we used the random forest algorithm (Breiman, 2001) to estimate the impurity importance of each SNP marker. This estimation was performed considering the Gini importance, which quantifies the normalized total reduction in the criterion (MSE) achieved by each feature (the sum of the feature importance across all markers is equal to 1). To obtain a more refined subset comprising only the markers most likely associated with agronomic traits, we established the major importance set by selecting the top 3 Gini importance markers in each phenotype clipping (**Figure 1**). In cases where the sum of these three values did not reach 0.5, we continued selecting additional values until the condition was satisfied. Furthermore, a PCA was performed using the major importance markers dataset.

### 2.5 Transcriptome assembly, quantification and annotation

Previous RNA-Seq data of 11 genotypes of *U. ruziziensis* were used to assess gene expression (Hanley et al., 2021; NCBI BioProject PRJNA513453). Raw data were quality-trimmed using Trimmomatic v0.39 (Bolger et al., 2014). The Illumina adapters, the first 12 bases of the read, and the leading and trailing bases with quality less than 3 were trimmed; the sliding window of 4 bases was set to cut the read when quality/base was less than 20 and only reads with more than 75 bases were kept. Then, the filtered reads were *de novo* assembled by Trinity v2.5.1 (Grabherr et al., 2011) considering a minimum contig length of 300 bases, and assembly integrity was evaluated using the Trinity.pl package utility (**Figure 1**).

SALMON 1.1.0 (Patro et al., 2017) was used to quantify transcript expression, which was subsequently summarized at the gene level using the tximport R package (Soneson et al., 2015). We retained only genes with more than one transcript per million (TPM) in at least three of the 11 samples, disregarding genes with low-level expression. The longest isoform for each gene was selected, and BUSCO v5.2.2 (Manni et al., 2021) was used to evaluate the annotation completeness against the Viridiplantae database. Finally, we aligned the filtered assembly to the UniProt database (Bateman et al., 2020) using Blastx and Blastn 2.10.0 (Altschul et al., 1990) with an e-value cutoff of 1e-10. Gene Ontology (GO) terms were retrieved using Trinotate software (Bryant et al., 2017), which performed functional annotation (**Figure 1**).

### 2.6 Genes linked with markers and GO enrichment

To identify genes physically linked to major importance markers (section 2.4), we conducted alignments between the genes derived from the transcriptome assembly (section 2.5) against the *U. ruziziensis* genome (Pessoa-Filho et al., 2019). Therefore, genes that aligned in a window of 5,000 bp up- and downstream of the marker position were considered physically linked. The alignment was performed using Blastn 2.10.0 (Altschul et al., 1990) with a minimum query coverage of 75% and an E-value cutoff of 1e-6. To visualize the gene position within the genome, we constructed a physical map using MapChart v2.32 (Voorrips, 2002), including information regarding the phenotype and the seasonal associations, as well as Gini importance (**Figure 1**). In addition, a circular map was constructed using the R package circlize v0.4.14 (Gu et al., 2014) to show the associated genes that were duplicated.

Finally, to obtain a functional profile of the genes linked to the markers, biological process GO term enrichment analysis was performed. This step was achieved with the R package topGO (Alexa and Rahnenfuhrer, 2022), and GO terms with p values< 0.01 in Fisher’s exact test were considered significantly enriched.

### 2.7 Coexpression network

We modeled a GCN using the transcript quantifications normalized in transcripts per million (TPM) and the highest reciprocal rank (HRR) (Mutwil et al., 2009) approach, considering a limit of 30 edges. From the GCN, we selected the genes associated with the agronomic traits and included highly correlated genes that were not considered in the network ranking (Pearson correlation coefficient ≥ 0.9 and a maximum p value of 0.01 with Bonferroni correction). From this defined gene set, we selected the first and second gene neighbors in the GCN. To evidence the gene associations with the two seasons, we highlighted genes related to phenotype clippings 2,3,7,8 and 9, considering them as components of a wet-season associated network. Similarly, genes associated with clippings 4, 5 and 6 were selected to form the dry-season associated network.

Network visualization and evaluation were performed using Cytoscape software v3.9.1 (Shannon et al., 2003). For each gene, we calculated the degree centrality measure with the methods of Barabási and Oltvai, 2004, and considered the genes with outlier values as hubs. Finally, biological process GO term enrichment analyses were performed for the selected genes, including first and second neighbors, to produce a general and seasonal functional profile of the metabolic pathways associated with the agronomic traits with the same method described in 2.6 (**Figure 1**).

## 3 Results

### 3.1 Phenotypic and genotypic data analyses

In our study, we evaluated five important traits for forage grasses (GM, DM, RG, LDM, and STM) across various clippings selected based on wet and dry seasons. Individual measurements were averaged at the subfamily level, and we excluded data from the first clipping. The descriptive analysis of subfamily based phenotypic data did not reveal any discernible patterns concerning the dispersion and skewness of the traits (**Supplementary Figure S2**). We did not identify any outliers in 17 out of the 33 traits. Despite the absence of any apparent similarity in phenotypic dispersion between the phenotypes evaluated, the correlation analysis yielded significant values for all the comparisons conducted (**Supplementary Figure S3**). We observed an average R Pearson correlation coefficient of 0.72 (**Supplementary Figure S3**), with the strongest correlations (~1) observed between the same clippings of GM and DM. Additionally, early clippings (2 and 3) tended to be less correlated with all other measures. This pattern was particularly more pronounced for GM, DM, and SDM. In contrast, SDM in clipping 2 exhibited the lowest correlation with all other phenotypes (**Supplementary Figure S3**). The progeny mean narrow-sense heritabilities for all phenotype clippings showed a mean value of 0.79, ranging from 0.44 (SDM in clipping 2) to 0.92 (LDM in clipping 5) (**Supplementary Table S1**).

The GBS experiment generated ~720 million reads, which were processed into 1.3 million tags using the Tassel pipeline. We identified a total of 77,413 SNP markers in this step. After applying quality filters, estimating allele frequencies, and imputing missing genotypes, we retained 28,106 of these markers. This final dataset of markers is referred to as the “complete data” (CD).

By using the phenotypic and genotypic data, we performed PCAs, plotting the dispersion of subfamilies using the scores of the first two principal components (PCs) (**Supplementary Figures S4**, **S5**). Although arising from different sources of variation (the proportion of variance explained by the first two PCs was 85.2% and 57.2% for the phenotypic and genotypic data, respectively), similar patterns could be observed. To corroborate such a similarity, we colored the samples from the genotypic PCA scatter plot using PC1 of the phenotypic data. Even without a pronounced presence of 3 groups, as in the phenotypic PCA, the coloring in the genotypic PCA evidenced a clear association between both PCA results (**Supplementary Figure S5**).

### 3.2 Genome-wide family prediction

The predictive performance of the GP models at the family level using the CD was assessed through the consideration of two conventional approaches (RKHS and BRR) across 33 phenotypes. Employing a 100-times 5-fold CV strategy, the RKHS model exhibited slightly superior results compared to BRR, with a mean PA of ~0.762 and mean MSE of ~0.025, contrasted to a mean PA of ~0.745 and a mean MSE of 0.026 in BRR. We observed a maximum PA of ~0.875 in the DM-8 trait and a minimum PA of ~0.490 in SDM-2. Aiming to achieve higher performance levels, we evaluated four ML algorithms (SVM, RF, AB and MLP). Among these models, SVM exhibited the best overall performance, with a mean PA of ~0.759 and a mean MSE of ~0.026; however, it did not surpass the performance of the RKHS approach. By considering Tukey’s test results for MSE, it became evident that the RKHS model significantly outperformed SVM, emerging as the superior approach in 30 traits compared to 13 of SVM (**Supplementary Tables S2**-**S4**). Our results indicate that when using CD for prediction, the ML algorithms were unable to outperform the performance of conventional models.

To increase our predictive accuracy and assess potential associations between traits and markers, we selected specific subsets of SNPs for each of the 33 traits based on the intersections established between FS sets. Each FS approach yielded a distinct quantity of markers: FS-1 selected sets with quantities ranging from 129 to 175 markers (mean of ~150, 0.53% of the CD); FS-2 from 484 to 1154 (mean of ~848, 3% of the CD); and FS-3 from 563 to 853 (mean of ~699, 2.5% of the CD). By considering the intersection approaches established, we obtained FI-1 with SNP quantities ranging from 76 to 122 markers (mean of ~102, 0.36% of the CD) and FI-2 with quantities varying from 5 to 23 markers (mean ~11, 0.04% of the CD) (**Supplementary Table S5**). In addition to obtaining more restricted sets, these markers selected by FI have more evidence of trait associations, as they were selected by multiple algorithms. In this sense, model performances using the CD were contrasted with the use of models created from the datasets selected by FI-1 and FI-2.

The employment of the FI datasets increased the performance of all models for all traits. This improvement was particularly pronounced in the AB and RF models, which presented the highest levels of accuracy, overcoming RKHS in both FI sets. Among the six models evaluated, the FI-1 approach presented an improved overall performance when compared to FI-2, being considered by Tukey’s test the best approach in 168 (FI-2 = 100) and 136 (FI-2 = 89) scenarios for PA and MSE, respectively (**Supplementary Table S6**). However, individual results for the best models in each scenario were similar, as indicated by the best model in FI-1 (AB with a mean PA of ~0.894 and a mean MSE of ~0.013) and FI-2 (RF with a mean PA of ~0.893 and a mean MSE of ~0.013) (**Supplementary Tables S2**-**S4**). Furthermore, when analyzing the clippings of a phenotype, we observed that the best performances for clippings in the combinations AB-FI-1 and RF-FI-2 varied in GM and DM, but for RG (clipping 3), SDM (clipping 5) and LDM (clipping 5), the results were equivalent (**Supplementary Tables S2**-**3** and 7).

In this sense, we observed that for the prediction task, both combinations AB-FI-1 and RF-FI-2 can be employed with comparable performance levels. However, for investigating trait−marker associations and catalogs of putative associated genes, FI-2 represents a more restrictive approach. With sets (mean of ~11 markers) approximately ten times smaller than the sets of FI-1 (mean of ~102 markers), FI-2 markers provide a group of markers with a probable reduced number of false positive associations. Therefore, we considered the combination RF-FI-2 as the most promising approach to be employed in our datasets. In addition to the significant decrease in marker density through FI-2, the RF algorithm demonstrated high efficiency for prediction with a PA increase of 6.9% and an MSE reduction of 22.6% when compared to the RKHS using the FI-2 dataset or 17% when compared to the RKHS using the CD dataset (**Table 1**).

**Table 1:** Comparison of RKHS and RF model predictive ability and mean squared error for all phenotype clippings using the FI-2 datasets.

Phenotype	Clipping	Predictive ability				Mean squared error			
		RKHS	RF	Diff.	Diff. (%)	RKHS	RF	Diff.	Diff. (%)
Green Matter	2	0.857	0.850	-0.007	-0.8%	0.018	0.018	0	0.0%
	3	0.870	0.869	-0.001	-0.1%	0.016	0.017	0.001	6.3%
	4	0.894	0.912	0.018	2.0%	0.015	0.013	-0.002	-13.3%
	5	0.866	0.917	0.051	5.9%	0.016	0.012	-0.004	-25.0%
	6	0.863	0.903	0.040	4.6%	0.018	0.012	-0.006	-33.3%
	7	0.844	0.923	0.079	9.4%	0.019	0.010	-0.009	-47.4%
	8	0.881	0.913	0.032	3.6%	0.020	0.015	-0.005	-25.0%
	9	0.897	0.944	0.047	5.2%	0.012	0.008	-0.004	-33.3%
	T	0.867	0.937	0.070	8.1%	0.018	0.009	-0.009	-50.0%
	Mean	0.871	0.908	0.037	4.2%	0.017	0.013	-0.004	-24.6%
Regrowth	2	0.868	0.896	0.028	3.2%	0.015	0.014	-0.001	-6.7%
	3	0.940	0.956	0.016	1.7%	0.011	0.008	-0.003	-27.3%
	4	0.859	0.911	0.052	6.1%	0.017	0.012	-0.005	-29.4%
	5	0.826	0.869	0.043	5.2%	0.016	0.013	-0.003	-18.8%
	6	0.833	0.884	0.051	6.1%	0.024	0.016	-0.008	-33.3%
	7	0.862	0.896	0.034	3.9%	0.015	0.011	-0.004	-26.7%
	8	0.860	0.872	0.012	1.4%	0.014	0.013	-0.001	-7.1%
	9	0.813	0.843	0.030	3.7%	0.017	0.014	-0.003	-17.6%
	T	0.886	0.932	0.046	5.2%	0.013	0.008	-0.005	-38.5%
	Mean	0.861	0.895	0.035	4.1%	0.016	0.012	-0.004	-22.8%
Dry Matter	2	0.537	0.638	0.101	18.8%	0.044	0.035	-0.009	-20.5%
	3	0.802	0.840	0.038	4.7%	0.014	0.013	-0.001	-7.1%
	4	0.935	0.941	0.006	0.6%	0.010	0.009	-0.001	-10.0%
	5	0.882	0.895	0.013	1.5%	0.014	0.015	0.001	7.1%
	6	0.884	0.917	0.033	3.7%	0.016	0.011	-0.005	-31.3%
	7	0.849	0.913	0.064	7.5%	0.017	0.011	-0.006	-35.3%
	8	0.911	0.915	0.004	0.4%	0.016	0.016	0	0.0%
	9	0.787	0.925	0.138	17.5%	0.022	0.011	-0.011	-50.0%
	T	0.912	0.943	0.031	3.4%	0.013	0.009	-0.004	-30.8%
	Mean	0.833	0.881	0.048	6.5%	0.018	0.014	-0.004	-19.8%
Leaf Dry Matter	2	0.835	0.875	0.040	4.8%	0.019	0.014	-0.005	-26.3%
	5	0.899	0.924	0.025	2.8%	0.012	0.011	-0.001	-8.3%
	T	0.900	0.952	0.052	5.8%	0.011	0.007	-0.004	-36.4%
	Mean	0.878	0.917	0.039	4.5%	0.014	0.011	-0.003	-23.7%
Stem Dry Matter	2	0.664	0.818	0.154	23.2%	0.033	0.021	-0.012	-36.4%
	5	0.904	0.921	0.017	1.9%	0.011	0.012	0.001	9.1%
	T	0.694	0.835	0.141	20.3%	0.025	0.015	-0.010	-40.0%
	Mean	0.754	0.858	0.104	15.1%	0.023	0.016	-0.007	-22.4%
	Overall Mean	0.839	0.892	0.052	6.9%	0.018	0.013	-0.004	-22.6%

### 3.3 Major importance markers

Given that the FS strategies employed in our study relied on ML algorithms estimated through a combination of decision trees, and that the top-performing models for FI-1 and FI-2 were AB and RF, respectively, we employed an additional approach to assess marker−trait associations using decision tree structures. We ranked the markers based on RF scores obtained from the FI-2 selected markers. We selected the top three Gini importance markers for each trait, and if the sum of importance for these top three markers did not reach at least 0.5 (out of a total of 1.0), we continued selecting markers from the ranking until we reached half of the total importance score. This process allowed us to compile a list of markers with the highest feature importance, thus preventing underrepresentation of importance across traits. From the 283 FI-selected markers across the 33 traits, we identified a subset of 69 markers with significant predictive relevance. Notably, only for SDM clipping 5, we had to select four markers instead of three (**Supplementary Table S8**).

Furthermore, we performed a PCA to evaluate the subfamily dispersion considering this set of 69 major importance markers. The first two PCs explained 67.5% of the data variance, an intermediate value between the complete set of SNPs (57.2%) and the phenotypic data (85.2%) (**Supplementary Figure S6**). Although the values of the first PCs seem to be inverted in such a PCA when compared to the others performed, we observed a similar dispersion pattern (**Supplementary Figures S4**, **S5**). As we expected, the scatter plot displayed a group formation visually closer to the phenotypic PCA. Since the markers were selected through associations with the traits, there was a strong relation between the major importance data PC1 and the samples colored using the phenotypic PC1 values (**Supplementary Figure S7**).

To assess the physical distribution of the FS-selected markers, we constructed a physical map for *U. ruziziensis* using the values obtained from the species’ genome. In addition to the set of 69 major importance markers, we incorporated all the FI-2 markers into the constructed map (**Figure 2**). Regarding the distribution of these markers, we observed associations across all chromosomes without a clear pattern, except for the presence of extensive regions with little or no markers, primarily located in the central regions of chromosomes 1, 2, 5, 7 and 8. We speculate that these regions correspond to the centromeric regions (**Figure 2**). Chromosome 1 presented the highest number of associations when considering both FI and major importance marker sets, with relatively consistent representativeness. However, it was also the chromosome with the highest number of identified SNPs (**Table 2**). On the other hand, chromosomes 5 and 6 presented the lowest presence of associations, while chromosome 4 experienced a significant change in representativeness, with a 7% reduction from FI to major importance (**Table 2**). Furthermore, especially in chromosomes 1, 4 and 7, we observed regions characterized by a high density of minor importance markers near major importance markers, which may suggest the presence of QTL regions associated with agronomic traits.

**Table 2:** Number and percentage of SNP markers identified/selected in each chromosome considering the complete data (CD), feature intersection (FI-2) and top Gini importance datasets.

Chromosome	Complete Data	Feature Intersection - 2	Top Gini Importance
**1**	4722 (16.8%)	69 (23.4%)	16 (23.2%)
**2**	3565 (12.7%)	36 (12.2%)	10 (14.5%)
**3**	3249 (11.6%)	28 (9.5%)	9 (13%)
**4**	3552 (12.6%)	42 (14.2%)	5 (7.2%)
**5**	1384 (4.9%)	15 (5.1%)	4 (5.8%)
**6**	2508 (8.9%)	23 (7.8%)	2 (2.9%)
**7**	3796 (13.5%)	37 (12.5%)	8 (11.6%)
**8**	2127 (7.6%)	18 (6.1%)	8 (11.6%)
**9**	2426 (8.6%)	22 (7.5%)	5 (7.2%)
**Scaffolds**	777 (2.8%)	5 (1,7%)	2 (2.9%)
**Total**	28106 (100%)	295 (100%)	69 (100%)

The major importance set was composed of various markers associated with more than one trait. As evidenced in the physical map, the marker associated with more trait clippings is on chromosome 7, position 42,826,434. This marker was associated with four of the five phenotypes evaluated and was selected for nine clippings, three of which had a Gini importance higher than 0.4 and in six Gini importance between 0.2 and 0.4 (**Figure 2**). Other markers were associated with various trait clippings, such as a marker on chromosome 1 position 69,834,400, which was associated with six trait clippings, and three other markers with four associations in chromosomes 1 and 3 (**Figure 2**).

When evaluating the markers for each of the five traits without separating them by clippings, we analyzed the intersections of sets to quantify markers associated with multiple traits (**Supplementary Figure S7**). Despite the variation in marker quantities between the FI-2 and major importance sets, the logical relationships among the trait sets remained consistent: GM, DM, and LDM shared the highest number of markers, while RG and SDM had a higher proportion of exclusive markers. Interestingly, SDM and RG exhibited generally lower correlations with the other traits as well.

### 3.4 Marker genes associated with phenotypes

To obtain a set of genes expressed by the species and subsequently assess their coexpression, we employed a previously published transcriptome of 11 *U. ruziziensis* genotypes. The sequencing of the libraries produced a total of ~1.7 billion reads, with 95.5% (**Supplementary Table S9**) being retained and used for *de novo* assembly. The resulting transcriptome encompassed 575,524 transcripts, of which 223,593 were categorized as unigenes, featuring a transcript N50 length of 1,227 bp. Following filtration based on expression levels, the dataset was reduced to 288,487 transcripts, representing 49,445 unigenes. The evaluation of assembly completeness was performed by comparing the 49,445 unigenes against the Viridiplantae database. From the 425 total BUSCO groups searched, we found 297 complete sequences (69.8%), 48.2% as a single copy and 21.6% as duplicated copies, in addition to 74 (17.4%) and 54 (12.8%) fragmented and missing sequences, respectively.

In the process of functional annotation, we aligned the transcripts to the UniProt database and obtained 197,045 associated GO terms. Among these, 6,156 were unique GO terms. This collection of genes and GO terms was then employed to perform a biological process GO term enrichment analysis of the genes linked to the major importance markers.

After aligning transcripts with the reference genome of *U. ruziziensis* and considering a window of 5,000 bp up/downstream of the marker positions, we mapped a total of 217 genes (264 considering genes with multiple copies) in close physical proximity to 58 markers (**Figure 2** and **Supplementary Table S8**). We did not detect genes linked to all markers, such as on chromosome 1, where no genes were found within a marker region associated with six traits clippings, or on chromosome 5, where out of the four major importance markers, two lacked associated genes (**Figure 2**).

**Figure 3:**
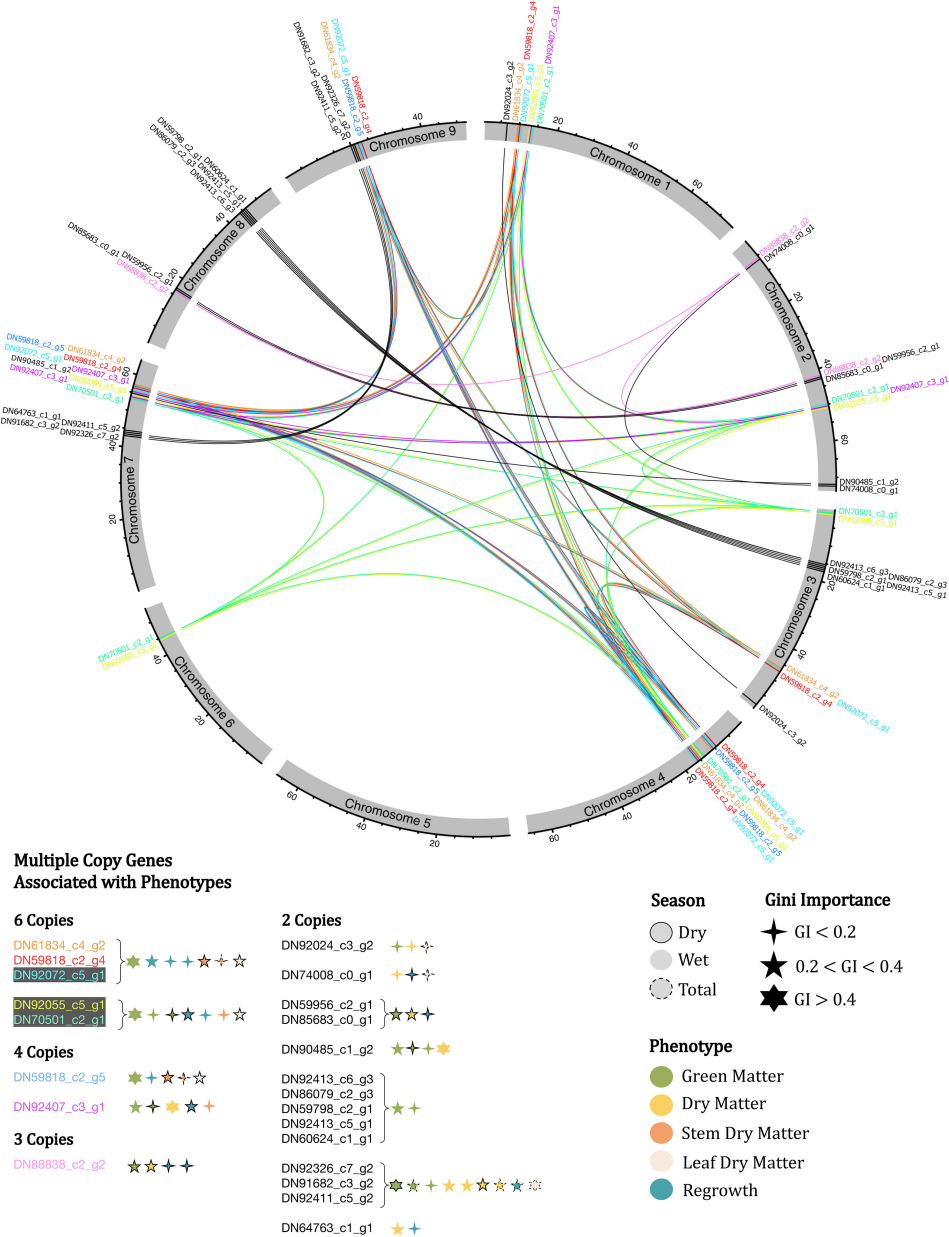
Circular map of the *U. ruziziensis* genome, indicating multiple copy genes identified as associated with the phenotypes evaluated, indicating Gini importance and season. The same genes are indicated with the same color, except genes with two copies.

As previously stated, we identified genes with multiple copies that are linked to more than one major importance marker region. There were 22 genes meeting this criterion, and they are highlighted in red in **Figure 2**. To facilitate a more comprehensive investigation of these genes, we represented their distribution in a circular map that illustrates their genomic positions. Additionally, we combined information about copy number variation, trait/season associations, and Gini importance (**Figure 3**). Among these genes, we identified five genes with six copies. Notably, three of these genes are found together, and collectively, they are associated with seven different trait categories. Furthermore, we identified genes with 4, 3 and 2 copies, all linked to all the evaluated traits, albeit with varying levels of importance, demonstrating no clear pattern.

Regarding the functional annotation of the genes associated with the phenotypes, we identified proteins/enzymes and GO terms for 100 of the 217 genes (**Supplementary Table S8**). In the region associated with more traits, on chromosome 7 (position 42,826,434), seven annotated genes were mapped, some of which were cinnamoyl-CoA reductase 1, and DEAD-box ATP-dependent RNA helicase 25. Furthermore, on chromosome 1 (position 11,178,365), which is associated with four traits, there are genes annotated to the multidomain protein RHM2/MUM4 which is involved in UDP-D-glucose to UDP-L-rhamnose conversion (**Supplementary Table S8**). Considering the genes with multiple copies, only three had functional annotation, which translates into AIM25-altered inheritance rate of mitochondria protein 25, cinnamoyl-CoA reductase 1 and E3 ubiquitin-protein ligase SINAT5.

Beyond specific protein annotation, to obtain a general functional profile of the proteins identified, we performed an enrichment analysis of the biological process GO terms and obtained a profile with 18 significant terms (p value< 0.01). The enrichment analysis identified terms associated with various phenotype clippings, such as “lignin biosynthetic process”, “auxin efflux” and “flavonol biosynthetic process” (**Supplementary Table S10**).

### 3.5 Coexpression network

To provide deeper insights into the functional patterns of genes associated with the agronomic traits evaluated, we modeled a GCN using the gene quantifications from the *U. ruziziensis* accessions. From a total of 49,445 genes, we identified significant interactions between 14,141 genes, represented as nodes in the network structure, connected by 17,812 edges (**Supplementary Figure S8**). Within this GCN, we found 54 genes from the 217 genes associated with the major importance markers. As we restricted the GCN created to the top 30 gene associations, we expanded the collection of 54 selected genes to more than 109 by considering correlations with a minimum Pearson coefficient of 0.9 and a Bonferroni corrected p value of 0.01. This group of 153 genes was considered directly associated with the traits evaluated.

**Figure 4:**
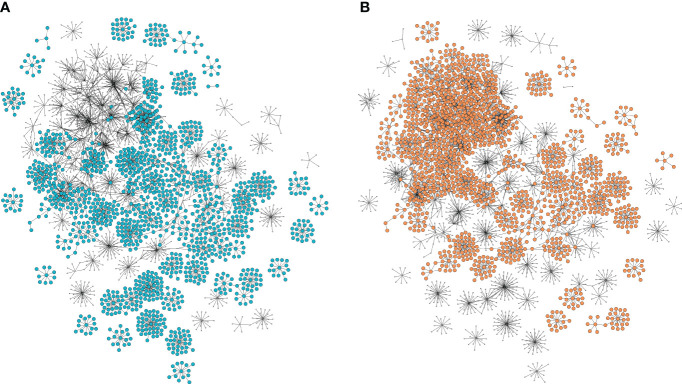
Selected and correlated gene coexpression network with first and second neighbors. Each node represents a gene, and each connection represents their correlation. **(A)** Genes associated with wet season trait clippings. **(B)** Genes associated with dry season trait clippings.

The potential of a GCN to elucidate metabolic pathways lies in its ability to identify genes that, despite not being selected by the prediction methodology, exhibit coexpression with them. To this end, we extended the set of 153 genes previously selected to the GCN first (308 genes) and second gene neighbors (2233 genes), creating a comprehensive agronomic trait network comprising a total of 2704 genes (nodes) and 3453 edges (**Figure 4**).

The functional profile of the general agronomic traits network was determined through an enrichment analysis of biological process GO terms, which revealed 11 significant terms (p< 0.01) for the gene set excluding the second neighbors and 16 terms when considering all genes in the network (**Supplementary Table S11**). When examining the restricted set, which excluded the second neighbors, we found enriched terms related to hormones, such as auxin efflux and abscisic acid transport, as well as biosynthetic processes involving molecules such as flavonoids. In the broader set that included second neighbors, we identified terms associated with DNA metabolism, including mismatch repair, DNA replication, and DNA duplex unwinding. Additionally, other enriched terms were linked to responses to stress, such as response to chitin and regulation of circadian rhythm.

To further explore the differences in functional gene patterns associated with the different seasons, we separated the general agronomic trait network into two seasonal parts. The genes associated with the traits in clippings 2,3,7,8 and 9 were selected for the wet season-associated network, and the genes associated with the traits in clippings 4, 5 and 6 were selected for the dry season-associated network. The wet and dry-season networks encompassed 33 and 22 genes associated with the major importance markers, 58 and 54 highly correlated genes, 102 and 231 first neighbors, 1322 and 1359 second neighbors, and a total of 1515 and 1666 genes (nodes) with 1801 and 2205 edges, respectively (**Figures 4A, B**). Comparing the seasonal functional profiles, we found shared terms such as flavonol biosynthetic process, auxin efflux and mitotic recombination-dependent replication fork processing. Additionally, we discovered season-specific terms such as abscisic acid transport, isoleucine biosynthetic process, response to nematode and chaperone-mediated protein folding for the wet season. In contrast, the dry season featured enriched terms such as pyridoxal phosphate biosynthetic process, response to water deprivation and response to chitin, all of which are related to stress response (**Supplementary Tables S12**, **S13**).

Another remarkable aspect of using GCNs to investigate the regulation of metabolic pathways lies in their ability to define hub genes, which possess a high number of connections in the network, as determined by the degree metric. The hub genes play an important role in regulating the functionality of numerous other genes, thereby potentially influencing the expression of the phenotypes that we are studying. In our modeled agronomic traits network, we found 235 hub genes (degree > 4), of which 14 had a degree > 40. Considering the seasonal networks, there were 107 and 158 hubs in the wet and dry season networks, respectively. Among the highest degree hub genes (>40), we found some present in both season networks, such as the genes that encode the 60S ribosomal protein L9 and the 14-3-3 protein zeta (**Supplementary Table S14**). While specific to the wet season, we found hub genes of the proteins ELF4-LIKE 4, SUV2 and lipid-transfer DIR1 (**Supplementary Table S15**) and to the dry season, 60S ribosomal L9, fatty acid-binding and 3-hydroxyacyl dehydratase FabZ (**Supplementary Table S16**).

## 4 Discussion

### 4.1 Genome-wide family prediction

Recent advances in omics approaches and computational methods for polyploid species have enabled the emergence of studies in important *Urochloa* breeding areas. These include genome assembly (Pessoa-Filho et al., 2019; Worthington et al., 2021), contaminant identification (Martins et al., 2021), transcriptomics (Vigna et al., 2016a; Salgado et al., 2017; Hanley et al., 2021; Jones et al., 2021; Worthington et al., 2021), linkage and QTL mapping (Ferreira et al., 2016; Thaikua et al., 2016; Vigna et al., 2016b; Worthington et al., 2016; Worthington et al., 2019; Worthington et al., 2021), GWAS (Matias et al., 2019b), and GS/GP (Matias et al., 2019a; Aono et al., 2022). Even with the recent progress, there are no studies employing integrative methodologies in the genus. Therefore, by leveraging the limited genetic resources available and employing biocomputational techniques, we have pioneered the first multiomic approach in the *Urochloa* genus, thereby expanding the molecular knowledge available for breeding.

In our initial approach to evaluating GWFP, we employed the complete marker dataset (CD) with conventional parametric and semiparametric GP models (BRR and RKHS). Our findings consistently yielded either higher or, at the very least, equivalent PA when compared to the achievements reported in other GWFP studies. While we achieved a high mean PA of ~0.8 for the DM clippings, in the case of alfalfa, the authors observed values below 0.7 for the same trait in both 10-fold and leave-one-group-out cross-validation scenarios (Murad Leite Andrade et al., 2022). Additionally, for ryegrass, an even lower PA value of 0.34 was observed in a leave-one-family-out cross-validation scenario (Guo et al., 2018). If we consider the GWFP results for other phenotypes, such as rust resistance and heading date in alfalfa (Murad Leite Andrade et al., 2022), as well as lignin content, stiffness and diameter in loblolly pine (Rios et al., 2021), they consistently exhibited smaller PA values compared to our results, which presented a mean PA of ~0.762 across the 33 traits. We attribute this PA in our predictions primarily to the relatively small population size and limited genetic diversity among our samples. This combination has been previously reported to enhance predictive accuracy, as demonstrated in wheat (Edwards et al., 2019). Moreover, increasing the population with genetically distant samples tends to increase the complexity of the prediction task, subsequently reducing its accuracy, as highlighted in previous studies (Lorenz and Smith, 2015; Berro et al., 2019).

The performance we achieved seems even more promising when compared to GP conducted at the individual level in tropical forages. Studies with *U.ruziziensis* interspecific hybrids (Matias et al., 2019a), *U. decumbens* (Aono et al., 2022), *M. maximum* (de C. Lara et al., 2019; Aono et al., 2022), and *P. virgatum* (Lipka et al., 2014) have yielded PAs ranging from values close to zero to maximum values of 0.7 when evaluating several agronomic and morphological traits and employing different cross-validation schemes. In addition to the well-known advantages of genomic selection, such as its potential to shorten the breeding process (Simeão-Resende et al., 2014) and reduce phenotyping costs (Crossa et al., 2017), the use of GWFP offers other advantages, particularly in forage breeding programs, which typically rely on family- or plot-level phenotyping for conventional breeding (Rios et al., 2021). Furthermore, when GWFP is conducted using ML models combined with FS/FI strategies, it has the potential to significantly lower genotyping costs.

Moreover, for the application of GWFP in breeding programs or for future research, we recommend prioritizing experimental designs featuring a greater number of families rather than increasing the number of individuals within each family. Studies with tetraploid full-sibling families have concluded that using six individuals is sufficient to effectively capture family variation, both in terms of genotyping and phenotyping (de Bem Oliveira et al., 2020; Rios et al., 2021). Furthermore, it is worth noting that to a certain extent, enlarging the training population holds the potential to enhance the performance of GWFP, as indicated by previous research (Fè et al., 2015).

The application of ML algorithms in GP has been extensively explored across various species and phenotypes (Grinberg et al., 2016; Lello et al., 2018; Liang et al., 2020; Chung et al., 2021; Islam et al., 2021; Sandhu et al., 2021). Although there is no concrete empirical evidence supporting the superiority of ML over linear methods (Zingaretti et al., 2020; Varshney, 2021), ML techniques have consistently demonstrated superior or at least equivalent performance compared to conventional models in diverse scenarios (Bellot et al., 2018; Abdollahi-Arpanahi et al., 2020; Liang et al., 2021; Wang et al., 2022). ML methods have the potential to outperform conventional GP models, especially when handling intricate phenotypes influenced by significant dominance and epistatic effects (Wang et al., 2018; Tong and Nikoloski, 2021). Moreover, there are no studies investigating the applicability of ML methods in GWFP. Thus, we evaluated four classical ML algorithms (SVM, RF, AB and MLP). Surprisingly, none of these algorithms was able to outperform the RKHS model. Although SVM demonstrated competitive performances for PA, it was not as good for MSE. RF and AB exhibited intermediate performances, whereas MLP markedly underperformed (**Supplementary Tables S2**-**4**). The poor performance of MLP may be attributed to the limited sample size of our dataset and the lack of hyperparameter tuning. Neural network methods are well known for their need for substantial datasets and meticulous hyperparameter tuning to achieve high prediction accuracy (Bellot et al., 2018; Montesinos-López et al., 2021).

### 4.2 Major importance markers

Our study goes beyond the applicability analysis of GWFP in *U. ruziziensis*. We also aimed to investigate the metabolic regulation of agronomic traits. As an initial step to achieve this objective, we aimed to establish potential marker-phenotype associations. To this end, the strong performance improvement observed using the FS/FI approach indicates that the selected sets of markers are likely to be near QTLs, and can therefore be used to define genomic regions involved in phenotypic variation (Steinfath et al., 2010; Heer et al., 2018; Zhou et al., 2019; Aono et al., 2020; Pimenta et al., 2021; Aono et al., 2022; Pimenta et al., 2022). Additionally, by utilizing allele proportions for genotyping the family, this approach can be extended to other crop species that employ the family as the unit for conducting GS.

In contrast to other approaches aimed at identifying genotype-phenotype associations, FS techniques do not rely on specific biparental populations (RILs, NILs, F2, etc.), which are necessary for QTL mapping (Mohan et al., 1997; Dhingani et al., 2015). Moreover, FS techniques have the ability to uncover nonlinear and complex associations, addressing a limitation of linear models used in GWAS (Korte and Farlow, 2013).

ML models based on decision trees offer good prediction interpretability since it is possible to assess feature importance. In the context of GP, these models can rank markers based on their association strength with the modeled phenotype (Azodi et al., 2019; Bayer et al., 2021; Medina et al., 2021). Therefore, given that the best model for each FI dataset type was equivalent, we computed the RF Gini importance for the more restricted FI-2 datasets and selected the most significant features to create an even smaller and more reliable set of putatively agronomic trait-associated markers. By using half-sibling families’ bulks as a representation of the genetic variability available for breeding, genotyping similar agronomic traits in various clippings and selecting only the most influential markers in the predictions, we were able to minimize the limitations of the method due to small sample size and obtain a reliable set of markers.

In this major importance set, we identified markers associated with multiple phenotypes. Notably, the number of shared markers was more pronounced for GM, DM and LDM, which is in accordance with the observed correlations among phenotype clippings, where SDM and RG exhibited lower correlations with the other traits (**Supplementary Figures S3**, **S6**). The high overall correlation, with a mean of 0.72, among traits and the overlap of the identified markers were as expected. This is because the assessed biomass characteristics are highly similar and likely influenced by the same metabolic processes. The DM phenotype was determined by drying the GM material, while SDM/LDM phenotyping involved separating the DM into stems and leaves. Furthermore, biomass production is dependent on the plant’s growth capacity (RG). Consequently, the narrow-sense heritabilities of these traits within the families were also very similar. As discussed in other studies, modeling performance is strongly influenced by heritability (Wang et al., 2018; Xu et al., 2018; Murad Leite Andrade et al., 2022). Therefore, our prediction performances did not vary significantly and were correlated with the heritabilities (**Supplementary Tables S1**-**S3**).

In the absence of genome annotation, we employed RNA-Seq data in a multiomic approach to map genes physically associated with the major importance markers. Considering the similarity of the agronomic traits employed and the potential involvement of the same biological processes in their regulation, we then conducted a functional analysis that considered the annotation of all genes collectively. This allowed for an overview of the most influential processes governing biomass production and growth.

The enrichment of GO terms related to the annotated genes provides evidence of the methodological capacity to identify QTL regions influencing the evaluated agronomic traits (**Supplementary Table S10**). Associated with various phenotype clippings, terms related to the lignin biosynthetic process stood out. Previous research has established its significant impact on plant development (Yoon et al., 2015; Bahri et al., 2020). Mutants of lignin biosynthesis genes have shown phenotypes of dwarfism/reduced plant growth (Schilmiller et al., 2009; Li et al., 2009; Song and Wang, 2011), altered morphology (Elkind et al., 1990; Jones et al., 2001; Franke et al., 2002), and tissue browning (Bout and Vermerris, 2003; Xu et al., 2011; Saballos et al., 2012). Furthermore, terms associated with auxin efflux were identified, which are known for their importance in growth regulation. Auxin hormone effects depend on concentration and are primarily produced in meristematic and specific regions (Blakeslee et al., 2005). The transport and distribution of auxin within plant tissues constitutes an essential aspect of its function in plant organogenesis and morphogenesis (Woodward and Bartel, 2005). This transport is facilitated by influx and efflux carrier proteins, providing essential directional and positional cues for various developmental processes, including vascular differentiation, apical dominance, organ development, and tropic growth (Benková et al., 2003; Blancaflor and Masson, 2003; Friml et al., 2003; Blilou et al., 2005; Grieneisen et al., 2007).

Furthermore, the flavonol biosynthetic process, which is another enriched term identified in our results, is known to regulate plant growth and development by controlling auxin transport. Its effects are primarily observed in root elongation, quantity and gravitropic response (Jacobs and Rubery, 1988; Brown et al., 2001; Santelia et al., 2008; Grunewald et al., 2012). Flavonols can influence auxin transportation by different mechanisms, such as modulating the transcription of genes encoding auxin transport proteins (Peer et al., 2004), acting as kinase inhibitors that regulate the phosphorylation status of auxin transport proteins (Agullo et al., 1997; Peer and Murphy, 2007), or altering the cellular redox state (Fernández-Marcos et al., 2013). In addition, flavonols have antioxidant functions, acting in response to stress such as UV radiation, wounding, drought, metal toxicity, and nutrient deprivation. These conditions lead to the accumulation of reactive oxygen species (ROS), which can damage cellular components and consequently impact plant development (Winkel-Shirley, 2001; Baskar et al., 2018; Agati et al., 2020). The list of terms associated with plant growth, development and stress response continues with the folic acid biosynthetic process (Stakhova et al., 2000; Gorelova et al., 2017), galactolipid metabolic process (Jouhet et al., 2007; Kobayashi et al., 2007; Botté et al., 2011), and cellular response to cold.

The enriched terms provided an overview of the biological function of the identified genes. However, for the genes with multiple copies, limited information was generated, as only three out of the 22 genes had functional annotation. Nevertheless, these three genes appear to have a significant impact on the evaluated agronomic traits. One of these genes, DN91682_c3_g2 (cinnamoyl-CoA reductase 1), which was identified in two copies, is involved in the lignin biosynthetic process (Lauvergeat et al., 2001), circadian rhythm, and response to cold (Carpenter et al., 1994). The second gene, DN64763_c1_g1 (E3 ubiquitin-protein ligase SINAT5), also found in two copies, is known to play key roles in multiple plant developmental stages and several abiotic stress responses (Shu and Yang, 2017). Furthermore, although it has been reported in yeast, the gene DN92072_c5_g1 (AIM25-altered inheritance rate of mitochondria protein 25), which was found in six copies linked to major importance markers, acts in the cellular response to heat and oxidative stress (Aguilar-Lopez et al., 2016) (**Figure 3**) (**Supplementary Table S8**).

As a result of diverse mechanisms, such as whole-genome duplication, tandem duplication, and transposon-mediated duplication, plant genomes have an abundance of duplicated genes (Panchy et al., 2016). These duplicate copies can persist for several reasons: insufficient time for the accumulation of deleterious mutations or selection pressure to preserve redundant functions (Panchy et al., 2016). This pressure can arise from four mechanisms: gene dosage increase (Ohno, 1970), subfunctionalization (Force et al., 1999), gene balance (Freeling and Thomas, 2006), and paralog interference (Baker et al., 2013). Beyond identifying multiple copies of genes associated with agronomic traits, further investigation into the mechanisms influencing their retention and how these copies interact and impact the trait may provide valuable insights for improving breeding methods to achieve higher genetic gains.

### 4.3 Gene coexpression network

We conducted additional multiomic investigations to gain a deeper understanding of the metabolic pathways and regulatory mechanisms that govern the evaluated agronomic traits. We modeled a GCN and isolated the previously identified genes and their coexpressed neighbors (**Figure 4**). This integration has been successfully employed in different species and has produced noteworthy results (Calabrese et al., 2017; Schaefer et al., 2018; Yan et al., 2020; Francisco et al., 2021). The ability of such networks to simulate complex biological systems and uncover novel biological associations has transformed molecular biology research (D’haeseleer et al., 2000; Liu et al., 2020), enabling the exploration of regulatory relationships, inference of metabolic pathways, and transfer of annotations (Rao and Dixon, 2019). Following the “guilt-by association” principle, GCNs typically reveal interactions among genes with correlated biological functions (Oliver, 2000; Wolfe et al., 2005; Childs et al., 2011). Furthermore, this strategy can contribute to initiatives aimed at exploring targets for molecular perturbations, such as CRISPR. These inferences hold the potential to reveal genes capable of enhancing the loss or gain of functions, thereby influencing phenotypes relevant to breeding programs.

In this context, we could expand our set of identified genes through coexpression analysis, providing broader insight into the metabolic pathways influencing the observed phenotypes. Moreover, the annotated genes within these modules can serve as a basis to infer the biological functions of the unannotated genes. Our network modeling has extended our understanding of genes associated with the previously discussed enriched terms. It has also enabled the identification of new genes involved in biological processes related to DNA integrity, stability and metabolism. These genes act in mismatch repair, telomere capping, and duplex unwinding, all of which are known to impact the normal growth and development of plants to varying degrees (Tuteja, 2003; Kim and Kim, 2018; Karthika et al., 2020). Additionally, our network also expanded the genes involved in regulating the abscisic acid (ABA) transport. Modulating hormone levels within tissues and cells is critical for maintaining a balance between defense mechanisms and growth processes, especially in suboptimal environments. This regulation also plays an important role in controlling stomatal closure (Seo and Koshiba, 2011; Chen et al., 2020). Furthermore, the network has elucidated genes involved in regulating the circadian rhythm. Such a process not only allows plants to adapt to daily environmental changes but also enables them to anticipate and prepare for these challenges in advance (Millar, 2016; Kim et al., 2017; Creux and Harmer, 2019). Notably, the gene ELF4-LIKE 4, a key player in the circadian rhythm (Doyle et al., 2002), stands out as one of the hub genes with the highest degree value in the network (**Supplementary Table S12**). Finally, we also identified genes related to response to chitin, an important component of the plant immune system activated in the presence of pathogens such as fungi, arthropods, and nematode egg shells (Kombrink et al., 2011; Sánchez-Vallet et al., 2015).

Furthermore, by separating the general agronomic trait network into two seasonal parts, we were able to investigate the potential impact of metabolic processes on plant development and production during both wet and dry periods. In our findings, we identified enriched terms related to auxin efflux and flavonol biosynthetic processes in both networks. These results have already been discussed in the context of auxin transport regulation, indicating the importance of the hormone regardless of the season. During the wet season, in addition to the previously mentioned abscisic acid transport, we observed terms associated with plant development, such as the isoleucine biosynthetic process (Yu et al., 2013) and the response to nematodes, which are pathogens capable of modifying Plant Physiol., development, metabolism, and immunity (Eves-van den Akker, 2021). In contrast, within the dry network, we found enriched terms related to responses to water deprivation and protein transport. This provides evidence of the metabolic mechanisms required to deal with abiotic stress.

Network degree analysis provided a means to identify hub genes, which are the most highly connected genes in the network. These hubs typically encompass genes with broad regulatory functions or associations with essential roles in biological processes (Carlson et al., 2006; Reverter and Chan, 2008; Amrine et al., 2015). In our analysis, in addition to the previously mentioned ELF4-LIKE 4 protein, we identified several ribosomal protein genes as hubs, such as 40S S6 and S15a-2, 60S L9 and L14-2, 54S L12, and Ubiquitin-40S S27a-1. The heterogeneity of ribosome composition is well-known and forms the foundation of the specialized ribosome theory, which states that different groups of ribosomes are tailored to translate specific sets of mRNAs (Gilbert, 2011; Xue and Barna, 2012; Genuth and Barna, 2018). Although the major discussion in this field is concentrated in elucidating how changes in ribosome composition might facilitate the translation of specific groups of mRNAs (Norris et al., 2021), our results indicate another intriguing aspect of this theory. Although the precise connection between the observed hub ribosomal proteins and the translation of the genes linked to the hubs has yet to be established, we hypothesize that their coexpression may result from a coregulatory mechanism that ensures the availability of specific tailored ribosomes in sufficient quantities for translating the mRNAs of these linked genes. Regarding the relationship between ribosomal proteins and the characteristics evaluated in this research, it has been reported that *A. thaliana* mutants in these proteins are often smaller and have simplified/aberrant vasculature and polarity defects (Van Lijsebettens et al., 1994; Ito et al., 2000; Fujikura et al., 2009; Horiguchi et al., 2011), which can directly impact attributes related to regrowth and biomass production.

In addition, among the highest degree hubs in the network, we found genes associated with lipid metabolism. These specific genes encode important proteins, including the lipid-transfer protein DIR1, fatty acid-binding protein, 3-hydroxyacyl-ACP dehydratase, and 3-Ketoacyl-CoA Synthase 4. These proteins play roles in fatty acid biosynthesis (**Supplementary Table S14**). Fatty acids, which are common components of complex lipids, are reported to have important roles in plant biology, including cell structure and response to different stresses such as temperature changes (Routaboul et al., 2000; Iba, 2002; Hou et al., 2006), salinity, drought (Mikami and Murata, 2003; Gigon et al., 2004; Zhang et al., 2005), exposure to heavy metals (Verdoni et al., 2001; Chaffai et al., 2007; Maksymiec, 2007), and pathogens (Kachroo et al., 2003; Nandi et al., 2005). Fatty acids, as integral components of cellular membranes, suberin, and cutting waxes (Beisson et al., 2007), contribute to stress resistance by modulating membrane fluidity, releasing α-linolenic acid (Grechkin, 1998), serving as precursors for bioactive molecules (Hou et al., 2016), and acting as modulators of plant defense gene expression (Kachroo et al., 2003).

Another gene with broad activity identified as a hub is 2-oxoglutarate/Fe(II)-dependent dioxygenase (2-ODD) (**Supplementary Table S14**). This highly versatile enzyme facilitates numerous oxidative reactions, playing a crucial role in biosynthetic pathways for normal organismal function and the production of high value specialized metabolites (Farrow and Facchini, 2014). Its roles extend across various pathways, including DNA repair, histone demethylation, posttranslational modifications, auxin and salicylic acid catabolism, and biosynthesis of gibberellin, ethylene, flavonoid and glucosinolate. 2-ODD is reported to have a significant impact on plant growth and development (Farrow and Facchini, 2014).

Another important aspect of the methodology employed lies in its ability to identify regions associated with known genes linked to specific traits. Equally important is its capacity to elucidate unannotated genes that should be investigated. In our results, more than half of the identified genes linked to the major importance markers lacked functional annotation. Remarkably, some of these unannotated genes seemed to be highly important, as they were observed to have multiple copies and associations with various traits (**Figure 3**). When we expanded our analysis to the GCN, even more unannotated genes emerged, including important hub genes evidenced by their high degree values (**Supplementary Table S14**). These genes/regions are important targets to expand the knowledge on the metabolic regulation of agronomic traits and represent valuable information that can be applied in species breeding.

Our work is innovative in different aspects and represents a significant advance in the field of molecular breeding techniques applicable to tropical forages. This study marks the first exploration of the applicability of GWFP in a *Urochloa* species, being the first time that FS and ML algorithms have been employed in GWFP. These techniques not only enhance prediction metrics but also drastically reduce the number of makers required for accurate prediction. Furthermore, employing a multiomic approach, we integrated the selected markers with transcriptome data to construct a coexpression network capable of providing insights into the regulation of plant growth and biomass production in the species. The results demonstrate the great potential of molecular breeding in reducing breeding costs, expediting the release of new cultivars, and facilitating metabolic investigations, even in orphan species with high genomic complexity, such as tropical forages.

## Data availability statement

The datasets presented in this study can be found in online repositories. The names of the repository/repositories and accession number(s) can be found in the article/**Supplementary Material**.

## Author contributions

FM: Conceptualization, Formal analysis, Writing – original draft, Writing – review & editing. AA: Conceptualization, Formal analysis, Writing – original draft, Writing – review & editing. AM: Writing – original draft, Writing – review & editing. RF: Writing – original draft, Writing – review & editing. MV: Resources, Writing – review & editing. MP: Resources, Writing – review & editing. MM: Supervision, Writing – review & editing. RS: Conceptualization, Resources, Supervision, Writing – review & editing. AS: Conceptualization, Resources, Supervision, Writing – review & editing.
